# Irreversible Electroporation as a Valid Treatment Option for Hepatic Epithelioid Hemangioendothelioma: An International Multicenter Experience

**DOI:** 10.1007/s00270-024-03770-5

**Published:** 2024-06-06

**Authors:** Govindarajan Narayanan, Anthony Spano, Nicole T. Gentile, Michelle M. Shnayder-Adams, Varshana Gurusamy, David M. Levi, Breelyn A. Wilky, Ronald A. Mora, Raihan Noman, Praveen Peddu, Madelon Dijkstra

**Affiliations:** 1 Herbert Wertheim College of Medicine, Florida International University, Miami, FL USA; 2 Department of Interventional Oncology, Miami Cancer Institute, Baptist Health South Florida, Miami, FL USA; 3 Department of Interventional Radiology, Miami Cardiac and Vascular Institute, Baptist Health South Florida, Miami, FL USA; 4 Division of Vascular and Interventional Radiology, University of Michigan Hospital, Ann Arbor, MI USA; 5 Department of Interventional Radiology, MD Anderson Cancer Center, Houston, TX USA; 6 Division of Abdominal Transplant Surgery, Atrium Health Carolinas Medical Center, Charlotte, NC USA; 7 University of Colorado School of Medicine, Aurora, CO USA; 8 Department of Radiology, Rutgers New Jersey Medical School, Newark, NJ USA; 9 Department of Radiology, King’s College Hospital NHS Trust, London, UK; 10 Department of Radiology and Nuclear Medicine, Amsterdam UMC, Location VUmc, Cancer Center Amsterdam, Amsterdam, The Netherlands

**Keywords:** Hepatic epithelioid hemangioendothelioma (HEHE), Irreversible electroporation (IRE), mRECIST

## Abstract

**Purpose:**

Hepatic epithelioid hemangioendothelioma (HEHE) is a rare tumor with currently no established standard of care. This international multicenter retrospective study assesses the use of percutaneous irreversible electroporation (IRE) as an ablative tool to treat HEHE and provides a clinical overview of the current management and role of IRE in HEHE treatment.

**Material and Methods:**

Between 2017 and 2023, 14 patients with 47 HEHE tumors were treated with percutaneous IRE using CT-scan guidance in 23 procedures. Baseline patient and tumor characteristics were evaluated. Primary outcome measures included safety and effectiveness, analyzed using Common Terminology Criteria for Adverse Events (CTCAE) and treatment response by mRECIST criteria. Secondary outcome measures included technical success, post-treatment tumor sizes and length of hospital stay. Technical success was defined as complete ablation with an adequate ablative margin (intentional tumor free ablation margin > 5 mm).

**Results:**

IRE treatment resulted in technical success in all tumors. Following a median follow-up of 15 months, 30 tumors demonstrated a complete response according to mRECIST criteria. The average tumor size pre-treatment was 25.8 mm, accompanied by an average reduction in tumor size by 7.5 mm. In 38 out of 47 tumors, there was no evidence of local recurrence. In nine tumors, residual tumor was present. There were no cases of progressive disease. Median length of hospital stay was one day. Only one grade 3 CTCAE event occurred, a pneumothorax requiring chest tube placement.

**Conclusion:**

The current study provides evidence that IRE is a safe and efficacious minimally invasive treatment option for HEHE.

**Graphical Abstract:**

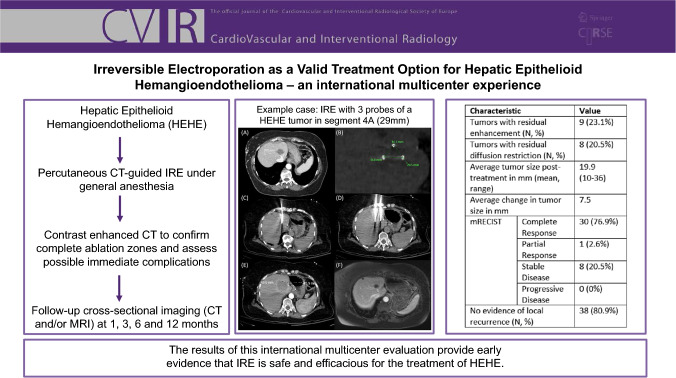

## Introduction

Hepatic epithelioid hemangioendothelioma (HEHE) is a rare vascular tumor with an incidence of < 0.1 per 100,000. Biologic behavior is variable, with many patients demonstrating stability of widespread metastatic disease for over ten years, but others with the potential for aggressive, unpredictable malignant transformation mimicking a high grade sarcoma [1]. HEHE has a heterogeneous clinical presentation and predominantly appears in the second to ninth decade of life, with a male-to-female ratio of 2:3 [1]. The underlying etiology is unknown, with several potential causes including, asbestos, oral contraceptives, thorotrast, vinyl chloride, primary biliary cirrhosis, viral hepatitis, hepatitis C and B, alcohol consumption, major hepatic trauma, and *Bartonella* infections [1–3]. The most common symptom is right upper quadrant pain in addition to hepatomegaly and weight loss, yet, a quarter of patients are asymptomatic [1]. The majority of patients presented with a multifocal tumor involving both lobes of the liver and no extrahepatic disease. However, it is not uncommon to see manifestations in the lung, bone, spleen, peritoneum, and/or local lymph nodes.

The diagnosis of HEHE can be difficult due to its nonspecific radiologic findings, and it is usually diagnosed incidentally via imaging [4]. The hallmark of HEHE on CT and MRI is peripheral rim enhancement of the vascular component of the tumor with a central composition of myxoid tissue that is devoid of enhancement [5]. HEHE may often be mistaken for cholangiocarcinomas or atypical hemangiomas, which present with similar patterns of enhancement on CT and MRI [5]. Despite advances made in imaging modalities to diagnose HEHE, biopsy and immunohistochemical evaluation is required for confirmatory diagnosis [4, 6]. In HEHE, invasion of (portal) veins is found, microscopically [7].

Since its discovery, HEHE has been managed with therapeutic strategies such as liver resection (LRx), liver transplantation (LTx), ablation and chemotherapy. LRx is often not appropriate due to the multifocal nature and the potential to develop extrahepatic manifestations. When unresectable, multiple systemic therapies have been utilized but are largely palliative in nature, with few meaningful long-term responses [2, 8–11]. Sangro et al. showed durable stabilization after two years of sorafenib with progressive calcification of the liver tumors in one patient [11].

Thermal ablation (i.e. radiofrequency ablation (RFA), microwave ablation (MWA) and cryoablation) has shown to be effective for HEHE in a recent study with a 5-year OS of 80% in six patients [12, 13]. A 3-year OS of 82% was found following transarterial chemoembolization in twelve patients [14]. Karaman et al. showed complete disappearance of FDG-uptake after Y90 transarterial radioembolization of HEHE in a case report [15]. Irreversible electroporation (IRE) has been successful in a diverse array of clinical applications [16]. Thomson et al. were the first to describe the successful application of IRE for HEHE [17]. Neal et al. published a case report of a 36 year old woman with HEHE tumors and a *Bartonella* infection treated with IRE and antibiotics [18]. At the time of the report, the patient remained stable for 38 months following treatment with no progression of disease or infection, and return of her serum liver enzyme levels to normal range. Another recent case of HEHE treated with IRE was detailed by Tong et al., in which the patient exhibited no local or distal progression of disease at 9 months post-procedure [19]. Despite the safe and effective nature of the procedure and success in these patients, there remains a need for further investigation of IRE in the treatment of HEHE in a larger cohort with longer follow-up. IRE may have an advantage over thermal ablation methods in the treatment of HEHE, as this primarily non-thermal technique can specifically target tumors near blood vessels due to the absence of the heat-sink effect and HEHE arises from epithelioid and histiocyte-like vascular endothelial cells [17, 20].

This multicenter retrospective experience and clinical overview aims to assess the use of IRE as an ablative tool to evaluate the safety and efficacy in the current management of HEHE.

## Methods

This international multicenter retrospective study was conducted at Baptist Health South Florida, Miami, Floria, United States of America and King's College Hospital NHS Trust, London, UK. Data reporting adheres to the ‘Strengthening the Reporting of Observational Studies in Epidemiology’ (STROBE) guideline [21]. This study has been approved by the institutional review boards.

### Patient Selection and Data collection

A retrospective chart review was performed on patients with biopsy proven HEHE, who underwent CT-guided IRE under general anesthesia. IRE was performed due to the proximity of the tumor to critical structures or vasculature. Patients who underwent IRE for HEHE with at least one follow-up imaging examination were included. Patients had sufficient kidney, liver, and bone marrow function and were medically fit to undergo general anesthesia. Cases were reviewed for baseline patient characteristics including age and gender and baseline tumor characteristics including specific location and staging.

### IRE-Procedures

All IRE-procedures were performed percutaneously with CT-guidance under general anesthesia. To evaluate changes in tumor size or characteristics and vasculature and critical structures in reference to the treatment trajectories, a pre-ablation contrast-enhanced CT was obtained. IRE is commercially available as NanoKnife™ (AngioDynamics), consisting of a generator, monopolar probes, and an AccuSync device, which is critical in synchronizing the generated pulses to the cardiac R-waves of the patient so as to avoid the potential risk of ventricular arrhythmia. Depending on the size of the treated tumors, 2 to 6 monopolar probes may be used to generate a maximum current of 3 kV. In this study, the choice of number of probes was based tumor size and placement of probes had a maximum distance of 2.3 cm. The probes were placed using CT-guided fluoroscopy followed by a 3D reconstruction and distance between probes in relation to tumor size to ensure proper placement of the probes. CT-fluoroscopy was used for intra-procedural guidance, and post-procedural imaging was performed with contrast-enhanced CT to confirm complete ablation zones (intentional tumor free ablation margin > 5 mm) and to asses for possible complications [22]. IRE was not offered in patients with a history of cardiac arrhythmias or lack of sufficient trajectory that may specifically interfere with the percutaneous technique of probe placement. Staged treatment was applied if a patient had more than three tumors. Repeat IRE treatment was offered if follow-up imaging showed partial response, stable or progressive disease according to mRECIST criteria [23].

### Imaging and Follow-up

Pre-procedurally, CT and/or MRI images were reviewed for tumor diameter, enhancement and diffusion restriction. Cross-sectional imaging (CT and/or MRI) at 1, 3, 6, and 12 months was used to assess treatment response, after which further imaging was obtained at the discretion of the medical oncologist.

### Primary and Secondary Outcome Measures and Statistics

Primary outcome measures were safety and effectiveness analyzed using Common Terminology Criteria for Adverse Events (CTCAE) 5.0, and treatment response was assessed using mRECIST criteria and SIO-DATECAN consensus document for time-to-event end points, respectively [23–25]*.* Secondary outcome measures included technical success, post-treatment tumor sizes, and length of hospital stay. Technical success was defined as complete ablation with an adequate ablative margin (intentional tumor free ablation margin > 5 mm) [22]. Baseline characteristics and procedural and tumor characteristics, as well as safety and efficacy outcomes, are presented as means for normally distributed data and as medians for non-normally distributed data, accompanied by ranges or expressed as counts and percentages of patients or tumors. Data analysis was performed using Excel (Microsoft), and results were tabulated.

## Results

### Patient, Procedure, and Tumor Characteristics

Fourteen patients (5 males and 9 females) underwent IRE from December 2017–August 2023. Average age at first treatment was 43.9 years (Table 1). A total of 47 tumors were treated with IRE in 23 treatment sessions, with median follow-up of 15 months (range 1–68) (Table 2). Two patients received staged procedures. Four patients were out of state and provided imaging reports as part of the follow-up, without post-treatment tumor sizes.

**Table 1:** Baseline patient characteristics (*N* = 14)

Characteristic	Value
Age in years (mean, range)	43.9 (25–71)
Sex	
Male (*N*, %)	5 (35.7%)
Female (*N*, %)	9 (64.3%)
Number of procedures (mean, range)	1.6 (1–4)
Number of tumors per patient (median, range)	3 (1–11)

**Table 2:** Procedure and tumor characteristics

Characteristic	Value
Number of treatments (*N*)	23
Previous treatment (*N*)	
Total	10
Systemic therapy	3
LTx/LRx	4
Ablation	2
Radiotherapy	2
Median follow-up (months, range)	15 (1–68)
Number of tumors per procedure (median, range)	2 (1–4)
Number of treated tumors (*N*)	47
Average tumor size pre-treatment in mm (mean, range)	25.8 (14–60)
Average number of electrodes used for IRE (median, range)	4 (3–5)

### Safety

Adverse events appeared in 14 out of 23 procedures (60.9%), with 13 minor (56.5%) and 1 major (4.3%) event. The most common adverse event was grade 1 or 2 abdominal pain occurring in 26.1% of cases. A CTCAE grade one or two hematoma occurred after five out of 23 procedures (21.7%). The hematomas were intraperitoneal, perihepatic, and subcapsular in location. No damage to critical structures, such as vasculature or bile ducts, was identified on immediate post-procedural imaging. One grade three CTCAE complication occurred, a pneumothorax requiring chest tube placement (Table 3). Median length of hospital stay was 1 day (range 1–3 days).

**Table 3:** Number of adverse events per procedure by Common Terminology Criteria for Adverse Events (CTCAE) Grading (25)

Adverse events	Minor (grade 1 or 2) (*N*, %)	Major (grade 3 or 4) (*N*, %)
Pain		
Abdominal	6 (26.1%)	0 (0%)
Non-abdominal	2 (8.7%)	0 (0%)
Hematoma	5 (21.7%)	0 (0%)
Atelectasis	2 (8.7%)	0 (0%)
Fever	2 (8.7%)	0 (0%)
Nausea and vomiting	2 (8.7%)	0 (0%)
Bloating	1 (4.3%)	0 (0%)
Extremity numbness	1 (4.3%)	0 (0%)
Itching	1 (4.3%)	0 (0%)
Pneumothorax	0 (0%)	1 (4.3%)

### Efficacy

**Fig. 1:**
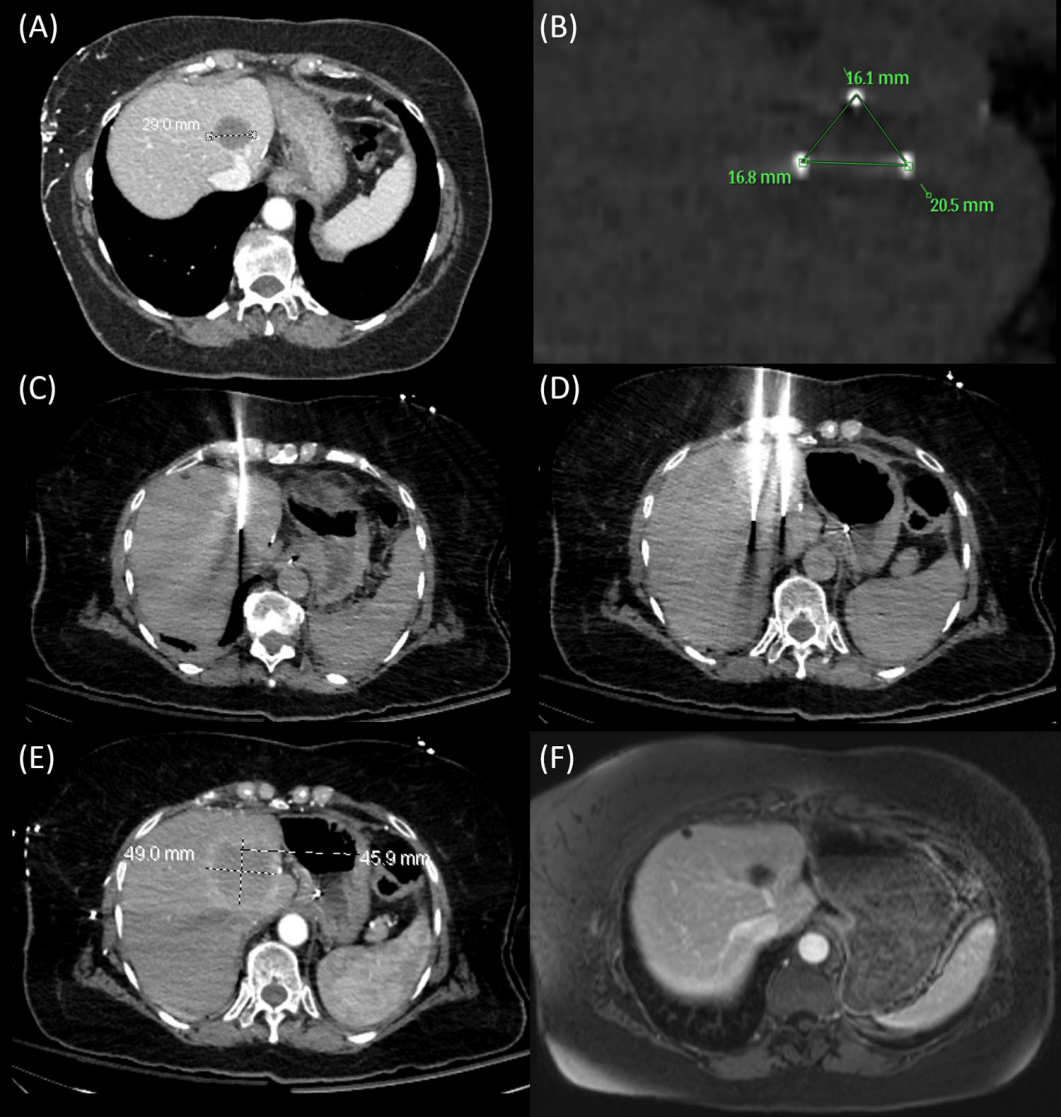
**A** Pre-procedural contrast-enhanced CT of the HEHE tumor in segment 4A measuring up to 29 mm. **B** Intraprocedural needle placement planning. **C** Intraprocedural CT showing intermittent placement of one out of three 17 gauge NanoKnife IRE ablation needles. **D** Intraprocedural CT showing intermittent placement of two out of three 17 gauge NanoKnife IRE ablation needles. **E** Intermediate post-procedural contrast-enhanced CT with an ablation zone measuring 46 mm × 49 mm. **F** Follow-up contrast-enhanced MRI after 8 months shows shrinkage of the ablation zone without residual enhancement

Technical success was achieved in all 23 procedures (100%). Treatment response is described in Table 4. A total of nine tumors had residual enhancement, of which eight had diffusion restriction at first follow-up. Following IRE, the average tumor diameter was 19.9 mm, with an average reduction in tumor size of 7.5 mm. The majority of tumors had complete response (*n* = 30) by mRECIST criteria, while one had partial response. Eight tumors were classified as stable disease by mRECIST, and there were no cases of progressive disease. In 38 tumors, there was no evidence of local recurrence at latest follow-up (80.9%). Four patients, with 6 tumors showing stable disease and one patient with one tumor showing partial response, all with concern for some residual disease, were successfully retreated. An example case of a successful IRE of one HEHE tumor is shown in Fig. 1.

**Table 4:** Treatment response

Characteristic	Value
Tumors with residual enhancement (*N*, %)*	9 (23.1%)
Tumors with residual diffusion restriction (*N*, %)*	8 (20.5%)
Average tumor size post-treatment in mm (mean, range)*	19.9 (10–36)
Average change in tumor size in mm*	7.5
mRECIST (23)*	
Complete response	30 (76.9%)
Partial response	1 (2.6%)
Stable disease	8 (20.5%)
Progressive disease	0 (0%)
No evidence of local recurrence (*N*, %)	38 (80.9%)

*Due to unavailability of radiological imaging, post-treatment tumor sizes were lacking in 8 tumors; however, radiological reports show that none of them had local recurrence

## Discussion

HEHE is an unpredictable tumor and can progress locally or develop metastatic disease. Research on epithelioid hemangioendothelioma in different organs is limited, and it is difficult to compare disparate patient populations [17]. The rare nature of the disease adds additional complexity in quality investigations. The present study evaluated the use of IRE for HEHE tumors in 14 patients. IRE treatment resulted in complete response of 76.9% of tumors by mRECIST criteria [23]. In 38 tumors out of 47, there was no evidence of local recurrence (80.9%). Tumors that were not adequately treated by one intervention were successfully treated with a second treatment. The average tumor size pre-treatment was 25.8 mm, with IRE treatment resulting in an average of 7.5 mm diameter decrease in tumor size through known apoptosis mechanisms. The study also demonstrates the safety of IRE with 14 (56.5%) minor adverse events and only one (4.3%) major adverse event (grade 3; pneumothorax requiring chest drainage). One grade 3 CTCAE event consisting of pneumothorax requiring chest tube placement [25].

Historically, there has been no standardized treatment for HEHE and various treatment modalities have been utilized over the past years. The two main surgical treatment options offered to treat HEHE include LTx and LRx. Mehrabi et al. described 286 patients with HEHE, and LTx was found to be the most common treatment performed [1]. Madariaga et al. described 17 patients treated with LTx with reported survival rates of 88%, 68%, and 59% at 1, 3, and 5 years, respectively [26]. Nudo et al. reported a 5-year survival rate of 82% for its 11 patients after LTx, but four patients developed recurrences within a median follow-up time of 15.6 months [27]. Due to inadequate numbers for statistical analysis, the efficacy of LTx in terms of mean survival rate remained uncertain. However, in selected cases, living donor LTx has been shown to be effective [28]. Despite some success in HEHE with LTX, Laüffer et al. study revealed a 5-year survival rate of 55.5% in HEHE patients regardless of treatment, thus further casting doubt on the necessity of LTx [29]. Therefore, the majority of cases in which LTx may be recommended involve diffuse spread of HEHE, with or without extrahepatic manifestations, and partial spread of HEHE with extrahepatic involvement [1]. Another concern with liver transplant is the requirement for lifelong immunosuppression which may limit eligibility for future clinical trials in the event of disease recurrence, as these patients are generally excluded.

LRx for HEHE is typically not recommended as HEHE typically manifest as multifocal if not diffuse in the majority of cases [1]. Although LRx has been successful in some case, it is still not the preferred modality of treatment given some cases of localized HEHE that exhibited aggressive behavior following LRx [30]. It has been hypothesized that the increase in reactivity from the resected tumor cells may contribute to an increase in activity of hepatotropic growth factors [2]. The therapeutic model suggested by Mehrabi et al. recommends LRx in cases of HEHE with partial liver involvement and absence of extrahepatic manifestations [1]. Ultimately, the decision to perform LRx is made on a case-by-case basis.

A broad collection of chemotherapeutic agents have been identified as effective treatment options, particularly in cases of nonresectable HEHE. Pazopanib is an antiangiogenic agent that specifically targets VEGFR-1/2/3, which are detectable on epithelioid hemangioendothelioma tumor cells [31]. The results were encouraging as indicated by the reduction in tumor size, stabilization of the tumor, and improvement in clinical symptoms. Oral thalidomide therapy is another antiangiogenic agent that was shown to successfully manage epithelioid hemangioendothelioma metastasized to the lungs [32]. Other antiangiogenic agents such as bevacizumab and sorafenib have shown promising results as well, the latter drug being administered orally for convenience and greater patient safety [11]. Interferon alpha-2B has been used as a successful adjunct therapy to LRx in the treatment of multifocal epithelioid hemangioendothelioma with metastatic potential [9]. These drugs are generally limited to adjunct therapy for LTx, as there is no substantial evidence to support their use in first-line management given limited sample sizes [2].

In the treatment of HEHE, IRE has an advantage over thermal ablation methods, due to the absence of the heat-sink effect [17]. IRE is a viable option for malignancies complicated by encroaching local blood vessels which would not be candidates for thermal ablation methods [33–35]. Given that HEHE cells have a tendency to proliferate alongside vascular structures, IRE is a promising treatment modality [36]. Furthermore, due to the minimally invasive nature, IRE carries benefit by not limiting other treatment options used concurrently or in the future [16].

An additional benefit of IRE is the healing mechanism post-treatment. Sugimoto et al. described the post-ablative differences in tissue healing between IRE and thermal ablation in primary liver cancer [37]. Thermal ablation techniques result in coagulative necrosis leading to fibrosis and scarring. After IRE, a reparative process can be observed with significant higher level of macrophage migration inhibitory factor compared to thermal ablation. This may potentially lead to immune stimulation, an early healing process, shrinkage of the ablation zone, and scarless tissue regeneration of the liver.

The present study has several limitations including its retrospective scope and non-comparative study design, data limited to two institutions, and relatively small sample size. Furthermore, the data were limited by a heterogeneous follow-up with a median of only 15 months and solely radiology reports in four patients. Another limitation was the relatively short term follow-up, though HEHE has a slow growing aspect. However, given that the incidence of HEHE is < 0.1 per 100,000, this study has a relatively large cohort to provide data on safety and efficacy of IRE in patients with HEHE.

## Conclusion

The results of this international multicenter evaluation provide early evidence that IRE is safe and efficacious for the treatment of HEHE. Additionally, HEHE is a rare disease frequently requiring multiple treatments for which IRE is a viable option, as it can be used for repeat treatments and has shown efficacy in hepatic tumors in proximity of critical structures. Future studies are required to further evaluate the long-term outcome of IRE for HEHE.
